# Exposure to Agrochemicals and Cardiovascular Disease: A Review

**DOI:** 10.3390/ijerph13020229

**Published:** 2016-02-18

**Authors:** Matome M. Sekhotha, Kotsedi D. Monyeki, Masezi E. Sibuyi

**Affiliations:** Department of Physiology and Environmental Health, School of Molecular Science and Agriculture, University of Limpopo (Mankweng Campus), Private bag X 1106, Sovenga 0727, South Africa; Kotsedi.monyeki@ul.ac.za (K.D.M.); eliot.sibuyi@ul.ac.za (M.E.S.)

**Keywords:** agrochemicals, farmworkers, cardiovascular system, blood circulatory system, ultrafine particles, particulate matter, nanoparticles

## Abstract

*Introduction:* In the agricultural world there is a continuous loss of food, fiber and other commodities due to pests, disease and weeds before harvesting time. These losses had create lots of financial burden to the farm owners that might lead to shutting down of their daily business. Worldwide, there is an overall very high loss of agricultural products due to weeds growth alone. To counteract this problem most farmers resort to the use of agrochemicals to increase their production but compromising the health of their farmworkers. The purpose of the study will be to assess the relationship between the agrochemical particles and cardiovascular diseases among farmworkers. *Method:* Non-systematic review was used to collect data. The following database were use: Medline, EBSCO, and Science Direct to search for the existing journal articles. *Results:* This study addresses the relationship between agrochemicals particles and cardiovascular diseases in the farming industries using literature review. *Discussion:* Other researchers had already done an extensive research on the pathway of potential mechanisms linking the ultrafine particulate matter to cardiovascular diseases. The outcomes of those investigations were the clinical results of events that might lead to the development of myocardial infarction, congestive heart failure (CHF), stroke, arrhythmia and sudden death. Xenobiotic compounds that maybe implicated in the pathophysiology of human cardiovascular diseases, will be examined and included in this study. There is compelling evidence suggesting that toxic free radicals of pesticides play an important role in human health. *Conclusion:* There is a close relationship between agrochemicals particle and cardiovascular diseases.

## 1. Introduction

In the agricultural world there is a continuous loss food, fiber and other commodities due to pests, disease and weeds before harvesting time. These losses had created lots of financial burden to the farm owners that might lead shutting down their daily business. The loss of agricultural commodities may reach a proportion of 25% in European countries due to uncontrollable weeds growth. Whereas, in less developed areas like Asia accounts of half potential products yield loss without the use of agrochemicals [1]. The loss of agriculture products plays critically important roles in the economy of many developing countries [2]. In order to counteract this loss the farmers cannot survive without the use of the agrichemicals in increasing their production yield and securing jobs for millions of farm workers and producing food for the world population.

The ultrafine particulate residue matter generated from the use of agrochemicals may pose health risks to farmworkers health. The numerous cohort studies have examined the relationship between long-term exposure of ambient fine particulate matter ≤2.5 μm (PM_2.5_) and non-accidental and cardiovascular mortality [3]. Cardiovascular disease called acute myocardial infarction (AMI) is a medical term for an event commonly known as a heart attack. An AMI occurs when blood stops flowing properly to the heart, and a consequent inadequate oxygenated blood supply might affect the heart muscle and proper functioning of the heart. This might be due to one of the coronary arteries that supplies oxygenated blood to the heart or from the body that develops a blockage due to unstable build-up of blood cells, cholesterol, lipids and particulate matter in blood pathway.

Currently, there have been many epidemiological studies illustrating the association between agrochemicals particles and various human body systems. These studies serve to fill the vacancy of epidemiological evidence in the comprehensive summary of environmental toxicology. The purpose of the study will be to assess the relationship between the agrochemical particles and cardiovascular disease among farmworkers. This article will provide a summary of subgroup of the results of cardiovascular risk from exposure due to agrochemicals among farmworkers. This will include the epidemiological studies, mechanisms of infiltration of agrochemicals particle in the heart and oxidative stress.

## 2. Methodology

### 2.1. Study Identification

This project is based on non-systematic approach method to review the existing literature. Publications articles that showed cardiovascular diseases due agrochemical exposure were identified through the following databases: PubMed, Science Direct and EBSCO search engines. A preliminary total of 150 related published research articles up to 2015 were selected using the various combination of keywords: “cardiovascular”, “agrochemicals”, “pesticides”, “herbicides”, “insecticides”, “farmworkers” with no restrictions of publication types and dates. The reference list of relevant publications identified were checked for additional studies and recent articles in relevant journals used to compile an adequate information for discussion. The whole search was limited to studies published in English in and peer reviewed journals only.

### 2.2. Criteria of Inclusion

The non-systematic review and identification of eligible studies were performed. The titles and abstract were screened to determine their relevance to the objective of the study. The full text of potentially relevant studies was then examined and applied eligibility criteria applied to the study (Table 1). Articles that were used the farmworkers must be healthy or without any prior medical conditions and non-cigarettes and alcohol drinkers.

Articles were considered eligible to review if fulfilled the following six inclusion criterion as shown in the Table 1.

## 3. Results

In study by Weichenthal *et al.* [4] statistical analysis hazard ratios (HR) and their 96% Confidence interval (CI) were estimated using Cox proportional hazard model. Three separate models (minimal, moderate and fully adjusted) were used to evaluate the relationship between ambient PM_2.5_ and non-accidental and cardiovascular mortality. Potential confounding factors included in the abovementioned models were added at different models. Simple models were examined without any confounding factors first-minimal model. This was followed by moderately models including potential important behavioural/personal factors, and finally adjusted models included socioeconomic and educational background. Sensitivity analyses resulted in stronger relationship between ambient PM_2.5_ concentration and cardiovascular mortality among men. There were significant positive association between PM_2.5_ and cardiovascular mortality in all three models for men as compared with women (Adjusted model-HR = 1.66; 95% CI: 1.04, 3.36; minimal model-HR = 1.32; 95% CI: 1.00, 1.76 and moderately model-HR = 1.41; 95% CI: 1.00, 2.02) (Table 2).

Each agrochemical concentration was log-transformed to improve the level of normality in a study conducted in United State. The adjusted concentration was used to calculate the association. For each agrochemical both lipid standardized and wet-weight concentration age, sex, race, education, family income, smoking, alcohol use, diabetes, serum cotinine and BMI were adjusted as potential confounders. Additional, logistic regression analyses were conducted to estimate the odd ratio of the agrochemicals in peripheral arterial disease (PAD) against subjects without PAD. Subjects with obesity added as confounder of PAD had significant increased mean lipids standardization value of p,p’-DDE (OR = 1.47; 95% CI: 1.08, 1.99) (Table 2). Most wet-weight concentration of agrochemicals also showed significant association that were similar to those of the lipid standardized concentration.

## 4. Discussion

### 4.1. Epidemiological Studies

A study that was conducted in Naivash which is situated 80 km North West of the Kenyan rift valley showed that most of the farmworker complain of health issues related to agrochemical. Most farms were green house, floriculture and horticulture with an extensive usage of wide variety of agrochemicals throughout the year to control the development of plants and weeds [6]. The large proportions of farmworkers were predominant spraying agrochemicals most of the time. Also, farmworkers that perform removal of weeds, planting and harvesting reported the highest proportion of symptoms potentially related to pesticide exposure over a period of time. The total number of 247 (34.5%) farmworkers showed cardiovascular symptoms (palpitation, chest pain and leg swelling) [6] (Table 2). There was an indication that a higher proportion of flower farm labours that continuously using the agrochemicals exhibited high incident of cardiovascular disease during their lifetime. These findings had been linked with handling and usage of agrochemical over time. The following: edema, pallor and tachycardia were some of the common symptoms related to cardiovascular disease of agrochemicals exposure. There was a total of 26 (23 males and 3 females) farmworkers responsible for spraying duties in the flower section. The total of 13 (50.0%) of these farmworkers complain of palpitation, chest pain and leg swelling. The mean comparison complainant and non-complainants of the spray user was significantly different with *p*-value < 0.05 [5] (Table 2). The over exposure to agrochemicals and health consequences needs to be taken into consideration by the farmers and government sectors to reduce the rate of mortality due to cardiovascular diseases.

A study conducted in North Carolina and Iowa reported that the use agrochemicals were positively associated with either myocardial infarction (MI) mortality or incidence among farm workers [4] (Table 2). Farmers were also exposed to several types of particulates matter, including diesel exhaust and high level of dust containing endotoxin and mold. A study in Sweden found an elevated risk of ischemic heart diseases mortality among those who experienced occupational exposure to due agrochemical exposure [8]. In North Carolina and Iowa the occurrence of mortality and incidence among farmworkers were due to agrochemicals as the risk factor for MI development.

The participants of older age, white race and those with family history of MI, cigarette smokers, obesity, hypertension and diabetes were also associated with an increased risk of MI (Table 2). Also alcoholic drinkers were more likely to experience MI if compared to nondrinkers [4]. Currently, there is an extensive literature that showed that confounders in Table 2 might have the negative impacts farmworkers. In all the study the baseline was established that to exclude the farmworkers with risk factors that might cause cardiovascular conditions. An ecological study in Montana, Minnesota and North and South Dakota found that those living in the countries with wheat production and exposed to agrochemicals were more likely to die from acute myocardial infarction over a period of time [9]. The pathophysiological mechanisms of particulate matter exposure and MI have been well characterized. Allon *et al.* [10] hypothesized that the pathophysiology of particulate matter (PM) occurs through an inflammation leading to the increase of plasma fibrinogen. There are lot of unreported case of dead or sick farmworkers due to agrochemical exposure without knowing the cause of that might cause death.

A study conducted in North Caroline and Iowa reported a total number of 839 non-fatal myocardial infarction among the 32,024 participants in the incidence analysis over a median 5.0 years follow-up period [8]. Fatal and non-fatal myocardial infarction cases were caused by the usage of individual agrochemicals over a period of time. The objective of that study was to assess the association as to whether agrochemicals applicators and other agricultural exposures to be related to fatal and non-fatal myocardial infarction among male and female pesticide applicators in Agricultural Health Study over a period of time. Of the 49 individual pesticides that qualify for both mortality and incidence analysis, 6 had been significantly positive associated with either cardiovascular mortality or incidence occurrence of agrochemical exposure [7].

In Table 3, a hazard ratio of more than 1 for one agrochemicals indicate that agrochemicals applicators more likely to be affected with a lethal dose of 50 (LD_50_).

Any agrochemical particles with the same active ingredients as mentioned above Table 3 has the chances of falling with the same 95% confidence interval range that might lead to health complications [11]. The worst agrochemical associated with mortality of male applicators was Ziram among agrochemicals spray applicators whereas dichlorodiphenyltrichloroethane showed an association with non-fatal (without detectable effect of mortality) myocardial infarction. No pesticide was associated with both mortality and non-fatal myocardial infarction. Table 3, illustrate that the impact of agrochemical can have on both gender lethal dose will depended on the exposure time. A study by Sriernstörm *et al.* [11] had reported that high prevalence of cardiac consequences among the farmworkers that apply agrochemicals over time. The most common agrochemicals used were organophosphate and carbamates herbicide.

Among female applicators acute toxicity of chlorphyrifos, coumaphos and cerbofuran pesticides vary from slightly to highly toxic that depends on exposure time. Chlorphyrifos, coumaphos and cerbofuran all inhibit acetylcholinesterase to varying degree which is responsible to monitor the heart beat [12]. An ecological study performed in Montana, Minesota and North and South Dakota found that those living in counties with high wheat production, use as a surrogate for herbicides exposure were more likely to die from acute myocardial infarction [9]. The relationship between agrochemical and health is of great interest in field of toxicology. A study in rats suggested that myocardial irregularities occur for up to 6 months after an acute high-level of exposure to a cholinesterase inhibitors [13]. The people with other cardiovascular confounders such as obesity, hypertension and stroke were likely to increase the exacerbation of health complications when exposed to agrochemicals over a period of time. The same results were found in South African whereby women were designated for soft labour [14].

Agrochemicals ultra-fine particle have the potentially to cause the peripheral artery disease (PAD) also known as peripheral vascular disease (PVD). The development of PVD is due to the narrowing of the arteries that supply the heart or brain by the presence of ultrafine particles or fatty acid [14]. The end results of this disease may include various form of infection or tissue death or coronary artery disease [15]. This blockage might be due to infiltration of ultrafine particle that can easily get stuck in the pathways of blood flow. The outcome of this blockage can be atherosclerosis [16]. The disease is associated with elevated cardiovascular morbidity and mortality in most countries [17,18]. In addition to the conventional risk factors, it had been shown that organochloride (OC) pesticides might be contributed to the development of PAD. Therefore, the confounders may increase the chances of cardiovascular diseases as stated in the previous paragraph. Also the recent studies using the National Health and Nutrition Examination survey (NHANES) data had shown a positive association between concentration of OC pesticides and self-reported cardiovascular disease among farmworkers [19].

Peripheral artery disease was linked to two body systems namely: integumentary and cardiovascular system. This might pose even a bigger challenge because intensive studies had been carrying out to show the close relationship between skin and agrochemicals. Whereby, integumentary system was of the route of absorbing agrochemicals particles. Therefore, individual farmworkers had more than one route of getting affected by the ultrafine particles. The end results will be in the blood circulatory system that might lead to the blockage of blood flow which will ultimately cause cardiovascular disease. 

Pollutants with aerodynamic diameter of PM_2.5_ and other confounders (hypertension, obesity, stress and smoking) had been generally associated with increased risks of myocardial infarction (MI), stroke, arrhythmia and heart failure exacerbation within hours of exposure in susceptible individuals [20]. The distribution of airborne ultrafine particulate matter due to agrochemicals particles and residual compounds have been linked with endothelial dysfunction and vasoconstriction that increase blood pressure prothrombotic and coagulant change, systematic inflammatory and oxidative stress response, autonomic imbalance and arrhythmia and progression of atherosclerosis. As consequence there was a significant relationship between agrochemicals residue with PM_2.5_. The ultrafine particles have the ability to penetrate and pass all different vessels until reaching the cardiac system to create blood flow blockage. The blockage of the blood flow to the target site of the heart might lead to development of other health conditions that might cause death due to cardiovascular disease (Table 2).

Therefore, most affected people will be farmworkers that work directly with agrochemicals in the field close proximity with the ground. Whereby, the other health effect of the blockage was unbearable caused by particulate to susceptible individual on the farm. The susceptible individual may the ones with other health conditions as shown in Table 2. Cardiovascular detrimental consequences due to ultrafine particles (UFPs) exposure had been observed in epidemiological studies (Table 2). Once agrochemicals particles deposited into the lung UFs in might gain the access to the blood circulation by different transfer routes and mechanisms, resulting in distribution throughout the body, including the brain [20]. The particulate matter (PM) is a uniquely important public issue amongst list of novel factors that might cause health complications. In particulate, particulate matter (PM) inhalation is one of the established trigger mechanism of cardiovascular events that might occurs within an hour to days of exposure depending on concentration level of agrochemicals particles. Moreover, the presence of agrochemicals ultrafine particle in the body system beyond serving a simple triggered that elicits numerous adverse biological responses have the potentially to cause death. This could likely be explained by translocation of UFPs from the respiratory epithelium migrating towards the blood circulation system and subsequent toxicity towards vascular endothelium. This might alter blood coagulation mechanism, triggering autonomic nervous system reflex action eventually alternating of the cardiac frequency and function mechanism of the cardiac muscle mechanism that might lead to cardiovascular disease. A survival analysis of in United State (US) Medicare data for a total number of 19,600 survivors of acute MI in 21 cities had shown the total risk of an adverse post MI outcome death [20]. This number was due to subsequent MI or first admission for congestive heart failure due to adverse increase exposure level of agrochemicals with PM_10_ concentrations over a period of time.

In general, findings from previous studies supported a causal relationship between chronic PM_2.5_ exposure and mortality. These relationships might cause the change in the biological mechanism including altered autonomic functions, impaired vascular function and increased pulmonary and systemic inflammation [21].

A study that was done in US showed the conversely, positive association between PM_2.5_ particles and cardiovascular system mortality in men over a period of time The agrochemicals with ambient PM_2.5_ was associated with 26%–28% increases in cardiovascular mortality in Harvard six cities [22]. The findings of previous study conducted by Weichenthal *et al.* [6]; Waggoner *et al.* [23] suggested that long-term exposure to ambient PM_2.5_ concentration may had adverse health effect among health population. The previous evidence also suggested a strong relationship between ambient PM_2.5_ cardiovascular mortality among women and men farmworkers [6,24,25].

### 4.2. Mechanism of Inflitration of Particulate Matter in the Heart

#### 4.2.1. Blood

The pathway of potential mechanisms that is, linking the ultrafine particulate matter and cardiovascular disease was outlined by Frankling *et al.* [26]. The end results will be clinical events that might lead to myocardial infarction, congestive heart failure (CHF), stroke, arrhythmia and sudden death. The infiltration of particulate matter in the blood circulatory system was through different pathways *i.e.*, ingestion, inhalation and dermal contact [24,27]. The presence of agrochemical particles in the blood circulatory system raises a concern for health researchers, scientists and health professions. The last places for the particulate matter will be either in the brain, heart, lung or liver. Accumulation of particles over period of time might lead to the obstruction of blood flow to sensitive areas in the human body. This obstruction mechanism of blood flow may lead to serious health complications which may ultimately cause death. Previous studies showed that pesticides exposure often induces acute and chronic human diseases [28]. A study conducted in China showed that the level of agrochemicals with PM_10_ in blood samples was high among farmworkers over time [29]. The same author found out that there were extensive long and short term health effects on Chinese farmer workers exposed to agrochemicals containing PM_10_. The high and medium toxic levels of agrochemical particles were most harmful to the farmworkers and their family members. A study by Gress *et al.* [5] also provided an extensive analysis of the critical role in which glyphosate-based herbicides markedly affect cardiovascular system in mammals.

#### 4.2.2. Oxidative Stress

Reactive oxygen species and their highly destructive nature have been known for at least 50 years. The devastating pathophysiology effects of oxidative stress due to agrochemicals ultrafine particulate matter on vital body organ are still of great interest to the researchers, scientist and health professions [30]. The xenobiotic compounds have the ability to produce free radicals that might lead to adverse health effect when inhaled or ingested or in contact with human skin. These free radicals from agrochemicals compounds maybe implicated in the pathophysiology of many human diseases. There is an evidence of an imbalance between the production of free radicals and the ability of the body to counteract or detoxify their harmful effect through neutralization by antioxidant that is an important mechanism of reducing the possibility of atherosclerosis development [30]. Agrochemical particles recognized as a potential hazard to the biological system. Particulate matter may lead to the palpation and/or blockage heart increasing the blood pressure that might lead to sudden death [5,6]. Some of agrochemicals might be indirectly affect the normal functions of the heart.

A study conducted in Argentina observed that the presence of high concentration levels of heavy metals in the blood sample of farmworkers was due to continuous usage of agrochemicals with metals as active ingredients [31,32,33]. This might be due to long exposure to having heavy metals to control aliens’ plants. Other laboratories had also demonstrated a positive correlation between plasma heavy metals and increased cardiovascular disease incidence, suggesting the elevated plasma level of ceruplasmin (CRP) that should be considered as a risk factor for coronary disease [31,32,33,34,35]. The involvement of xenobiotic-induced oxidative stress by PM_2.5_ agrochemicals is well known in the etiology of many human diseases by weakening the cardiac muscle that will affect the normal functioning of the heart and potentially lead [36].

## 5. Conclusions

The developments of agrochemical compounds toxic effect amongst farmworkers need further investigation. The amount of literature available that link the particle matter less or equals to particulate matter 2.5 to cardiovascular system need an adequate investigation. As state in the abovementioned paragraphs adverse effects of low dose PM_2.5_ from agrochemicals mixtures on the cardiovascular system is largely unknown. The agrochemicals with potential toxicity to human health will continue be used by farmworkers as long as the general population need to food to survive. But the health effects of agrochemicals have a serious catastrophic impact on human health. The need for more food place the farm workers to produces more fruits and vegetable by using agrochemical to keep up with the demands compromising their health status. The detrimental conditions in which the farmworkers are placed by the farm owners are highly affected by the unequal economic status of the world and labour exploitation worldwide. The underlying etiological mechanisms of agrochemical particulate matter and cardiovascular system need further investigation in relation to other confounders. It is clear that agrochemical have serious effects on human health especially on the cardiovascular disease which was the focus of this article. Also, the mechanisms of preventing usage of agrochemicals as well as their adsorption through the skin maybe another focus of the future research. This will help in understanding the PAD etiological development in relation with cardiovascular disease.

The wide variety agrochemicals used farmers as well as the prevalence of unreported cases of mortality due to cardiovascular system is of serious concern to the health profession which might not be the case for agricultural sectors. The prevalence of unreported case of mortality due to cardiovascular system is of serious concern. The number of farmworkers that use agrochemicals then retired due to ill health normally die without knowing the cause of their death. Instead most complain of wide variety of diseases and health conditions with the lack of clear evidence of the cause of their death. This is a common circumstance in undeveloped countries due to financial constrains that will able to conduct forensic pathological investigation of the deceased. The banning of agrochemical in the agricultural sectors will produce devastating economics effects and people will suffer worldwide. Therefore, different stakeholders in the agricultural and government sector must play a significant role to curb and decrease the agrochemical-cardiovascular condition in order to decrease mortality percentage.

## Figures and Tables

**Table 1:** Inclusion criteria for research articles to be used in compiling this non meta-analysis research.

Number	Criterion
1	It must be an original epidemiological study using a case-control, cross sectional prospective study design and other type of reviews
2	Paper should be written in English
3	The agrochemicals should be measured in actual blood samples
4	The reported measurement of association, include odds ratios (OD), and relative risk (RR) and confidence interval (CIs) for cardiovascular risk
5	Studies should use the biomarkers of any agrochemicals compounds used in the agricultural industries
6	Cardiovascular (CDV) was confirmed by self-reported questionnaires or hospital diagnoses

**Table 2:** Study characteristics.

Citation	Geographical Location	Sample Size	Study Design	Agrochemical Product of Exposure	Health Outcome Assessment	Reported Findings (OD, RR IM *etc.*)	Confounding Factors
Castaneda *et al.* [5]	Michigan	300	Cross sectional	Particulate matter	Cardiovascular system	Model 1: (obesity) OR 0.304, 95% CI: 0.246,0.889 * Model 2: (current smoking) OR 1.382,95% CI: 0.588, 3.249 * Model 3: (Hypercholesterolemia) OR 0.633, 95% CI: 0.207, 1.939 * Model 4: (Diabetes) OR 2.552 ,95% CI: 0.885, 7.359 Model 5: (Hypertension) OR 0.459, 95% CI: 0.155, 1.361 *	Hypertension;Obesity cigarette smoking; Type 2 diabetes; Hypercholesterolemia
Tsimbiri *et al.* [6]	Kenya	801	Cross sectional	Pesticides	Palpitations chest pain leg swelling	*p* value < 0.002	Malaise Headache Respiratory
Weichenthal *et al.* [4]	North Carolinea and Iowa	93,378	Retrospective cohort study	PM_2.5_ compounds	Cardiovascular most precise exposure	Minimal adjusted: HR 1.08, 95% CI: 0.75, 1.55 (All); HR 1.16, 95% CI: 0.77, 1.74 (Men); HR 0.84, 95% CI: 0.39, 1.82 (Women) Moderately adjusted: HR 1.25; 95% CI: 0.81, 1.94 (All) HR 1.52; 95% CI: 0.92, 2.51 (Men); HR 0.68; 85% CI: 0.28, 1.67 (Women) Fully adjusted: HR 1.31; 95% CI: 0.84, 2.04 (All) HR 1.66; 95% CI: 1.00, 2.78 (Men) HR 0.62; 95% CI: 0.25, 1.55 (Women)	Ischemic heart disease Cerebrovascular
Min *et al.* [7]	United State	31,126	Retrospective	p,p’DDE Trans-nonachlo Oxychlordane Diedrin Sum off five OC pesticides	Mortality by cause of death *p* < 0.05	Lipid standardized Obese OR 1.47; 95% CI: 1.08–1.99 Non-obese 0.92; 95% CI: 0.63, 1.34 Wet-weight (Without lipid adjustment) Obese: 1.52, 95% CI: 1.15, 2.03 Non obese: 0.92; 95% CI: 0.64, 1.30 Wet weight (With Lipid adjustment ) Obese: 1.56; 95% CI: 1.01, 2.44 Non-obese: 0.85; 95% CI: 0.54, 1.32	Diabetes mellitus
Mills *et al.* [8]	North Carolina and Iowa	54,069	Prospective Study	Carbamates, organochlorides organophosphates pyrethroids Herbicides Fumigants Fungicides	Myocardial infarction mortality	HR 1 (0–50 days) 0.76 (51–100 days); 95% CI 0.76–1.47, HR 1.06 *p*-value 0.93 (101–250); 95% CI 0.71–1.23 0.97 (>250 days); 95% CI 0.75–1.26 0.57 High exposure; 95% CI 0.57–1.38; HR 0.88	Family history of MI Smoking cigarette Obesity Hypertension Diabetes Alcoholic drinkers
					Myocardial infarction incidence	1 (0–50 days) 1.10 (51–100 days) 95% CI 0.83–1.46 1.14 (101–250) 95% CI 0.91–1.43 1.19 (>250 days) 95% CI 0.96–1.4 1.12 High exposure 95% CI 0.84–1.48	
					Myocardial infarction incidence	1 (0–50 days) 1.10 (51–100 days) 95% CI 0.83–1.46 1.14 (101–250) 95% CI 0.91–1.43 1.19 (>250 days) 95% CI 0.96–1.4 1.12 High exposure 95% CI 0.84–1.48	

* *p* < 0.1.

**Table 3:** The summarized association of pesticides usage and non-fatal myocardial infarction non-fatal incidence and mortality among male and female pesticides applicators, Agricultural Health Study, 1993–2006 [7].

Males			Women			
Pesticides	Hazard Ratio	Outcome	Agrochemicals		Odds Ratio	Outcome
Ethylene dibromide	1.54; 95% CI 1.05–2.27	Mortality	**Insecticides**	Chloropyrifos	2.1; 95% CI 1.2–3.7	Myocardial infarction
Maneb/mancozeb	1.34; 95% CI 1.49–3.86			Coumaphos	3.2; 95% CI 1.5–7.0	
Ziram	2.40; 95% CI 1.49–3.86			Carbofuran	2.5; 95% CI 1.3–5.0	
Aldrin	1.20; 95% 1.01–1.43	Non-fatal myocardial infarction	Herbicides	Pendimethalin	2.5; 95% CI 1.2–4.9	
Dichlorodiphenyltrichloroethane	1.24; 95% CI 1.04–1.46			Trifluralin	1.8; 95% CI 1.0–3.1	
2,4,5-trichlorophenoxyacetic acid	1.21; 95% CI 1.03–1.43		Fungicide	Metalaxyl	2.5; 95% CI 1.1–5.3	

## References

[1] Mańkowski J., Pudelko K., Kolodziej J., Karas T. (2015). Effect of herbicides on yield and quality of straw and homomorphic fibre in flax (*Linum usitatissimum* L.). Ind. Crop Prod..

[2] Xiao F., Pignatello J.J. (2015). Interaction of triazine herbicides with biochar Steric and electronic effects. Water Res..

[3] Beelen R., Hoek G., van den Brandt P.A., Goldbohm R.A., Fischer P., Schouten L.J., Jerrett M., Hughes E., Armstrong B., Brunekreef B. (1996). Long term effects of traffic-related pollution on mortality in a Dutch cohort (NLCS-AIR Study). Environ. Health Perspect..

[4] Weichenthal S., Villeneuve P.J., Burnett R.T., van Donkelaar A., Martin R.V., Jomes R.R., DellaValle C.T., Sandler D.P., Ward M.H., Hoppin J.A. (2014). Long term exposure to fine particulate matter: Association with non-accidental and cardiovascular mortality in the Agricultural health study cohort. Environ. Health Perspect..

[5] Castaneda S.F., Rosenbaum R.P., Hoischer J.T., Madanat H., Talavera G.A. (2015). Cardiovascular disease risk factors among Latino migrant and seasonal farmworkers. J. Agromed..

[6] Tsimbiri P.F., Moturi W.N., Sawe J., Henley P., Bend J.R. (2015). Health impact of pesticides on residents and horticultural workers in the lake of Naivash region Kenya. Occup. Dis. Environ. Med..

[7] Min J.Y., Cho J.S., Lee K.J., Park J.B., Park S.G., Kim J.Y., Min K.B. (2011). Potential role of organochlorine pesticides in the prevalence of arterial diseases in obese person: Results from National Health and Nutrition Examination Survey 1999–2004. Atherosclerosis.

[8] Mills K.T., Blair A., Freeman B., Sandler D.P., Hoppi J.A. (2009). Pesticides and myocardial Infarction Incidence and mortality among male pesticide applicators in the Agricultural health Study. Am. J. Epidemiol..

[9] Toren K., Bergdahl A., Nilsoon T., Järvholm B. (2007). Occupational exposure to particulate air pollution and mortality due to ischemic heart disease and cerebrovascular disease. Occup. Environ. Med..

[10] Schreinemachers D.M. (2006). Mortality from ischemic heart disease and diabetes mellitus (type 2) in four U.S. wheat producing states: A hypothesis-generating study. Environ. Health Perspect..

[11] Allon N., Rabinovitz I., Manistersky E., Mittleman M.A. (2001). Increased particulate air pollution and triggering of myocardial infarction. Circulation.

[12] Sriernstörm E.L., Holmberg S., Thelin A., Sväardsudd K. (2001). A prospective study of morbidity and mortality rates among farmers and rural and urban non farmers. J. Clin. Epidemiol..

[13] London N., Myers J.E., Nell V., Thompson M. (1998). Health status among farm workers in the Western Cape—Collateral evidence from a study of occupational hazard. S. Afr. Med. J..

[14] United States Environmental Protection Agency (2006). Organophosphorus Cumulative Risk Assessment—2006 Update.

[15] Allon A., Rabinovitz I., Manisstersky E., Weissman B.A., Grauer E. (2005). Acute and long lasting cardiac changes following a single whole-body exposure to sarin vapor in rats. Toxicol. Sci..

[16] Lloyd-Jones D., Adams R.J., Brown T.M., Carnethon M. (2010). Heart disease and stroke statistics—2010 update: A report from American Heart Association. Circulation.

[17] Fowkes F.G., Rudan D., Rudan I., Aboyans V., Denenberg J.O., McDermott M.M., Norma P.E., Sampson U.K., Williams L.J., Mensah G.A. (2013). Comparison of global estimates of prevalence and risk factors for peripheral artery disease in 2000 and 2010: A systematic review and analysis. Lancet.

[18] Criquii M.H., Deneberg J.O. (1998). The generalized nature of atherosclerosis: How peripheral arterial disease may predict adverse events from coronary artery disease. Vasc. Med..

[19] McDermont M.M., Liu K., Greenland P., Guralnik J.M., Criqui M.H., Chan C., Pearce W.H., Schneider J.R., Ferrucci L., Celic L. (2004). Functional decline in peripheral arterial disease: Association with the ankle brachial index and leg symptoms. JAMA.

[20] Gress S., Lemoine S., Seralinin G.E., Puddu P.E. (2015). Glyphosate-based herbicides potently affect cardiovascular system in mammals: Review of the literature. Cardiovasc. Toxicol..

[21] Goncharov A., Haase R.F., Santiago-Rivera A., Morse G., McCaffrey R.J., Rej R., Carpenter D.O., Akwesasne Task Force on the Environment (2008). A high serum PCBs are associated with elevation of serum lipids and cardiovascular disease in a Native American population. Environ. Res..

[22] Brook R.D., Franklin B., Cascio W., Hong Y., Howars G., Lipsett M., Luepker R. (2004). Air pollution and cardiovascular disease: A statement for Health care Professionals from expert panel on population and prevention science of the American Heart Association. Circulation.

[23] Brook R.D., Rajagopalan S., Pope C.A., Brook J.R., Bhatnagar A., Diez-Roux A.V., Holguin F., Hong Y., Luepker R.V., Mittleman M.A. (2010). Particulate air matter air pollution and cardiovascular disease: An update to the scientific statement from American Heart Association. Circulation.

[24] Laden F., Schwartz J., Speizer F.E., Dockery D.W. (2008). Reduction in fine particulate air pollution and mortlity extended follow-up of the Harvard Six Cities Study. Am. J. Respir. Crit. Care Med..

[25] Waggoner J.K., Kullman G., Henneberger P.K., Umbach D.M., Blair A., Alavanja M.C.R., Kamel F., Lynch C.F., Knott C., London S.J. (2011). Mortality in the Agricultural Health Study, 1993–2007. Am. J. Epidemiol..

[26] Miller K.A., Siscovick D.S., Sheppard L., Sullivan J.H., Anderson G.L., Kaufman J.D. (2007). Long term exposure to air pollution and incidence of cardiovascular events in women. N. Engl. J. Med..

[27] Frankling B.A., Brook R., Pope C.A. (2015). Air pollution and cardiovascular disease. Curr. Probl. Cardiol..

[28] Buckley N.A., Karalliedde L., Dawson A., Senanayake N., Eddleston M. (2004). Where is the evidence for treatments used in pesticide poisoning? Is clinical toxicology fiddling while the developing world burns?. J. Toxicol. Clin. Toxicol..

[29] Steenland K., Dick R.B., Howell R.J., Chrislip D.W., Hines C.J., Reid T.M., Lehman E., Laber P., Krieg E.F., Knott C. (2000). Neurologica function among termiticede applicators exposed to chloropyrifos. Environ. Health Perspect..

[30] Hu R., Huang X., Huang J., Li Y., Zhang C., Yin Y., Chen Z., Jin Y., Cai J., Cui F. (2015). Long and short term health effect of pesticides exposure: A cohort Study from China. PLoS ONE.

[31] Zarkovic N. (2003). 4-Hydroxynonenal as bioactive marker of pathophysiological processes. Mol. Aspects Med..

[32] Ellenhorn M.J., Schonwald S., Ordog G., Wasserberger J. (1997). Medical Toxicology: Diagnosis and Treatment of Human Poisoning.

[33] Arnal N., Cristall D.O., Tacconi de Alaniz M.J., Marra C.A. (2010). Clinical utility of copper, ceruplasmin and metallothionene plasm determine in human neurodegenerative patients and their first degree relatives. Brain Res..

[34] Arnal N., Astiz M., de Alaniz M.J.T., Marra C.A. (2011). Clinical parameters and biomerkers of oxidative stress in agricultural workers who applied coppers based pesticides. Ecotoxicol. Environ. Saf..

[35] Reunanen A., Knekt P., Aarn R.K. (1992). Serum ceruloplasmin level and the risk of myocardial infarction and stroke. Am. J. Epidemiol..

[36] Lopèz O., Hernández A.F., Rodrigo L., Gil F., Pena G., Serrano J.L., Parrón T., Villanueva E., Pla A. (2007). Changes in antioxidant enzymes in humans with long term exposure to pesticides. Toxicol. Lett..

